# One-and-a-half nostril versus binostril endoscopic transsphenoidal approach to the pituitary adenomas: A prospective randomized controlled trial

**DOI:** 10.3389/fsurg.2022.1007883

**Published:** 2022-09-23

**Authors:** Junhao Zhu, Guodao Wen, Chao Tang, Zixiang Cong, Xiangming Cai, Jin Yang, Chiyuan Ma

**Affiliations:** ^1^ Department of Neurosurgery, Affiliated Jinling Hospital, Medical School of Nanjing University, Nanjing, China; ^2^ Department of Neurosurgery, DongGuan SongShan Lake Tungwah Hospital, Dongguan, China; ^3^ School of Medicine, Southeast University, Nanjing, China; ^4^ Department of Molecular Cell Biology and Immunology, Amsterdam UMC, Vrije Universiteit Amsterdam, Amsterdam, Netherlands

**Keywords:** endoscopic surgery, pituitary adenoma, skull base, quality of life, olfaction

## Abstract

**Background:**

Binostril endoscopic transsphenoidal approach (BETA) is the most used approach for sellar lesions nowadays, while its damage to the nasal structures may cause nasal discomfort and affect nasal functions including respiration and olfaction. With the purpose to improve the post-operative sinonasal quality of life (QoL), we introduced the one-and-a-half nostril endoscopic transsphenoidal approach (OETA) in 2016 which preserved more natural structures and registered a prospective randomized controlled trial (ChiCTR-IOR-16008222) to compare the two approaches regarding the surgical outcomes and complications.

**Methods:**

Sixty patients with pituitary adenomas were recruited and randomly assigned to the OETA group and the BETA group between April 2016 and May 2017 in Jinling Hospital. The tumor resection rate, endocrinal and visual outcomes, and surgical complications between the OETA and BETA groups were analyzed. Besides, the questionnaire Anterior Skull Base Nasal Inventory-12 (ASK Nasal-12) was used to evaluate patients’ sinonasal QoL at seven time points (pre-operative; 2-weeks, 1-month, 3-months, 6-months, 12-months, and long-term post-operatively). The Sniffin’ Sticks were used to assess patients’ olfactory function objectively in a long term. Each patient was followed for at least 12 months post-operatively.

**Results:**

There was no significant difference in tumor resection rate, hormonal and visual outcomes, and surgical complications between the two groups. Regarding the ASK Nasal-12, patients in the OETA group complained less about dried nasal material at 2 weeks after surgery (*P* = 0.017). One month after surgery, the OETA group had better olfaction function (*P* = 0.019) compared with the BETA group. However, there was no significant difference in early and long-term postoperative sinonasal QoL between the two approaches according to the entire ASK Nasal-12 metric. The results of the Sniffin’ Sticks showed that the two groups had a similar olfactory performance at long-time follow-up.

**Conclusion:**

In this single tertiary center trial, the results showed that the OETA achieved the same surgical outcomes and post-operative sinonasal QoL as the BETA.

**Clinical Trial Registration:**

http://www.chictr.org.cn/showproj.aspx?proj=13852, identifier: ChiCTR-IOR-16008222

## Introduction

Pituitary adenomas (PAs) are the most common sellar lesions and account for approximately 15% of intracranial tumors (1). Its prevalence ranges from 77 to 115 cases per 100,000 individuals and is increasing over the past decades (2, 3). Patients with PAs may have symptoms including decreased visual acuity, visual field defect, headache, and tumor-associated endocrinopathies. Since fully endoscopic approaches to the sellar region were reported in the mid-1990s, the endoscopic endonasal transsphenoidal approach (EETA) has become the mainstay of treatment for PAs requiring surgical intervention (4).

The EETA is mostly operated through both nostrils (binostril endoscopic transsphenoidal approach, BETA), which requires the removal of the posterior nasal septum. Although it provides a wide view of the surgical field, its damage to the nasal structures may result in poor sinonasal quality of life (QoL) and anosmia (5–7). Another more minimally invasive approach, the mononostril technique, was also used in EETA. However, its working space was limited to one nostril (8).

In 2016, we introduced the one-and-a-half nostril endoscopic transsphenoidal approach (OETA) with the expectation of combining the advantages of the binostril and mononostril approaches (9). This approach provided superior exposure and surgical freedom and caused less damage to the nasal septum (10).

In this study, we reported the results of this prospective randomized controlled trial which was conducted to compare OETA with BETA in terms of surgical outcomes and complications.

## Methods

### Design of the clinical trial

**Figure 1:**
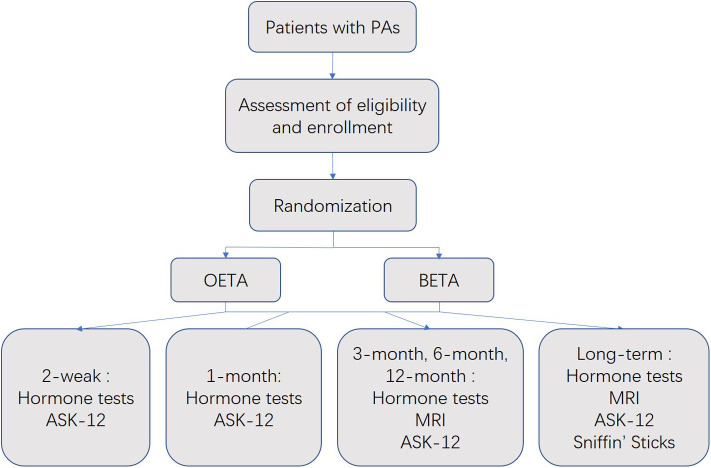
Flowchart of the trial. Anterior Skull Base Nasal Inventory-12, ASK Nasal-12; binostril endoscopic transsphenoidal approach, BETA; one-and-a-half nostril endoscopic transsphenoidal approach, OETA; pituitary adenomas, PAs.

The study was a prospective, randomized and controlled trial. This clinical trial (flowchart shown in Figure 1) was approved by the Ethics Committee of Jinling Hospital and registered in 2016 (ChiCTR-IOR-16008222). The study was carried out under the principles of the Declaration of Helsinki, local laws, and regulations. All patients gave informed consent to participate in this trial. Patient recruitment began in April 2016 and finished in May 2017.

The patients were eligible for enrollment if they were diagnosed as PAs by MRI and serum hormone assays, and surgical intervention was considered necessary by multidisciplinary experts. The patients with the following conditions were excluded from this study: (1) patients who have undergone endonasal surgery before; (2) patients with significant septal deviation which narrows the view and compromises the approach from the right side; (3) patients with other intracranial tumors; (4) patients with central nervous system infection or severe systematic infection; (5) patients with a history of chronic obstructive pulmonary disease, coronary heart disease, chronic kidney disease [GFR < 60 ml/(min * 1.73 m^2^)] and blood disorders; (6) women in the gestational or lactational period.

The primary outcome was the gross total resection (GTR) rate. The second outcomes include endocrine outcome, visual outcome, surgical complications, sinonasal QoL, and olfactory function. The trial was designed to show the non-inferiority of OETA to BETA regarding the primary outcome with a non-inferiority margin of −0.3. Accounting for a potential dropout rate, 64 patients (32 in each group) are needed to be enrolled, which ensured a power of 80%.

Sixty-four patients were enrolled and randomly (Simple Randomization) assigned to the two groups: the OETA group (OETA, *n* = 32) and the BETA group (*n* = 32). Four patients harboring PAs with significant lateral suprasellar extension that could not be adequately removed transsphenoidally were operated through EETA after randomization followed by craniotomy and excluded from this trial. At last, there were 29 patients in the OETA group and 31 in the BETA group.

The surgical procedures were described in detail in the following section. The patients were asked to return to the hospital for radiologic examinations and hormone level checks at 1, 3, 6, 12 months, and long-term points (more than 16 months) post-operatively for assessment of tumor resection rate and hormonal outcomes.

The criteria for the determination of hormonal remission vary depending on the tumor type. For prolactinoma, the criterion is a normal serum prolactin level. For growth hormone-secreting adenoma, the criteria are a normal value of IGF-1 and a suppression of GH excretion of less than 1.0 ng/ml during an oral glucose tolerance test.

The questionnaire Anterior Skull Base Nasal Inventory-12 [ASK Nasal-12 (11), shown in supplemental Table 1] was used to evaluate their sinonasal QoL at 7 time points (preoperative; 2-weeks, 1-month, 3-months, 6-months, 12-months, and long-term post-operatively). We also used Sniffin’ Sticks to assess patients’ olfactory function objectively at long-term points. Sniffin’ Sticks is an objective test of olfactory performance based on pen-like odor-dispensing devices (12). We did the odor identification test which is composed of sixteen multiple forced choices from a list of four descriptors (13). It has been validated and used in endoscopic skull base surgery to evaluate patients’ olfaction (14–16).

**Table 1:** Patient basic characteristics and pre-op symptoms.

	OETA (*n* = 29)	BETA (*n* = 31)	*P* value
Age	49.38 ± 13.98	50.90 ± 10.43	0.633
Male/Female	15/14	16/15	0.993
The course of the disease (month)	29.98 ± 33.64	16.49 ± 31.08	0.112
Duration of follow-up (month)	20.45 ± 4.86	21.03 ± 4.02	0.613
Maximum diameter (cm)	2.37 ± 1.10	2.43 ± 1.10	0.953
Knosp grade, *n* (%)			0.678
0	2 (6.90%)	1 (3.23%)	
1	5 (17.24%)	10 (32.26%)	
2	6 (20.69%)	5 (16.13%)	
3	11 (37.93%)	9 (29.03%)	
4	5 (17.24%)	6 (19.35%)	
Hardy grade, *n* (%)			0.821
0–2, A–B	16 (55.17%)	18 (58.06%)	
3–4, C–E	13 (44.83%)	13 (41.93%)	
Functioning adenoma, *n* (%)	7 (24.14%): 4 GH-secreting adenoma, 3 prolactinoma	4 (12.90%): 2 GH-secreting adenoma, 2 prolactinoma	0.261
Hypopituitarism, *n* (%)	4 (13.79%)	5 (16.13%)	0.800
Visual defect, *n* (%), *n* (%)	8 (27.59%)	12 (38.71%)	0.361
Headache, *n* (%)	14 (48.27%)	11 (35.48%)	0.260

The tumor resection rate, hormonal and visual outcomes, and surgical complications were confirmed at the latest follow-up and analyzed between the OETA and BETA groups.

### Blinding

The patients were not blinded while the data collection and statistical analysis were blinded for objective assessment.

### Surgical procedures

#### OETA

**Figure 2:**
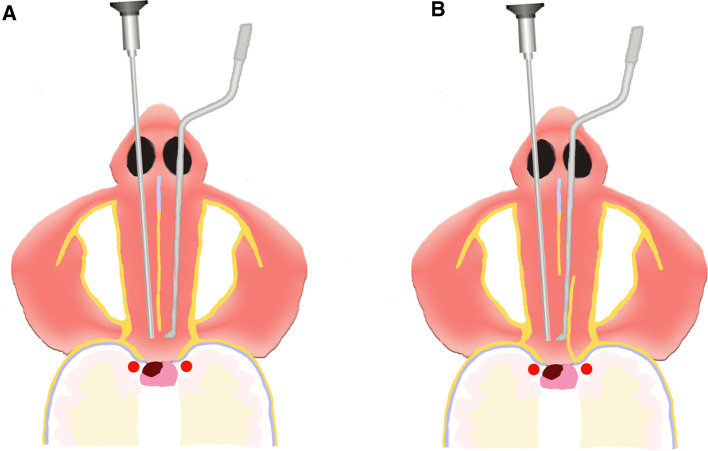
The difference between the OETA and BETA. (**A**) For BETA: a part of bilateral posterior septal mucosa was necessarily resected. (**B**) For OETA, the left mucosa of the posterior nasal septum was intact. As to the left nasal septal mucosa, only a 2 cm vertical incision was needed at the anterior level of the middle turbinate.

We performed OETA as we described before (9). Briefly speaking, the patient was under general anesthesia and in a supine position with 10 degrees of extension. The bilateral nasal cavities were packed with cottonoids containing 0.01% epinephrine for vasoconstriction and irrigated with iodine for disinfection. The operation started from the right nostril under a 0° endoscope (Karl Storz, Tuttlingen, Germany). The right inferior and middle turbinates were out-fractured for access to the sphenoethmoidal recess. Then, the right rescue flap was made with caution to protect the olfactory epithelium (17). The next operation was to dissect the bony nasal septum and expose the sphenoid rostrum. The left nasal septum mucosa was then pushed towards the left cavity to make the left sphenoid ostium visible. The main difference between the BETA and OETA was that the two approaches followed different methods of resecting the posterior nasal septum mucosa (Figure 2). For the BETA, a part of bilateral posterior septal mucosa was necessarily resected for bilateral access to the sellar pathology. For the OETA, the left mucosa of the posterior nasal septum was intact. As to the left nasal septal mucosa, only a 2 cm vertical incision was needed at the anterior level of the middle turbinate. The sphenoid ostium was enlarged with a low-speed drill and rongeur. After the enlargement of the sphenoid sinus and removal of the intra-sphenoid septum, the following procedures were the same as the BETA.

#### BETA

The nasal preparation and creation of the rescue flap were identical to the OETA. Sphenoidotomy was performed on both sides. A posterior septal window was created by removing the posterior part of the bony nasal septum to allow bilateral access. The optic nerve canal and the carotid prominence were identified as landmarks. The sellar floor was then flattened with a drill and opened with the rongeur. After opening the dura with scissors, the tumor was removed with curettes and a suction cannula. The 30°-angled endoscope was introduced into the sella for inspection of tumor remnants. If an intraoperative cerebrospinal fluid (CSF) leak occurred, the rescue flap was then fashioned to ensure a vascularized repair. Gelfoam and Tabotamp fibrillar (Johnson / Johnson Medical GmbH) were routinely used for skull base reconstruction. Nasal packing was not used routinely.

### Statistic analysis

The SPASS (IBM SPASS Statistics 26) was used for statistical calculation. Descriptive statistics were used to summarize patients’ demographic, clinical, and other outcomes. Continuous variables were assessed for normality and equality of variances between groups. Discrete variables were summarized by frequencies/proportions. For continuous variables, analysis of variance will be used, where appropriate. The comparison of the two groups concerning frequencies/proportions will be performed using the *χ*^2^ test and, if necessary, Fisher’s test. The ranked data of the two groups will be compared using the Wilcoxon rank sum test.

## Results

### Patient characteristics

A total of 60 patients were included in the trial (OETA: *n* = 29; BETA: *n* = 31). The average age of the OETA group was 49.38 (range 12–74) and for BETA it was 50.90 (range 20–69). All patients who participated in the trial received at least 12 months of follow-up and 51 of them received a longer observation which lasted for at least 16 months. The mean duration of follow-up was 20.45 months (range 12–31 months) in the OETA group and 21.03 months (range 12–28 months) in the BETA group.

All the PAs in the two groups were macroadenomas (maximum tumor diameter >10 mm), except for 2 microadenomas in the OETA group and 1 microadenoma in the BETA group. The mean maximum diameter was 2.37 cm for the OETA group and 2.43 cm for the BETA group. The invasive PAs (Knosp grade 3 and 4) accounted for 55.17% (16/29) in the OETA group and 48.38% (15/31) in the BETA group.

Concerning the endocrinological symptoms, 7 patients (3 prolactinomas and 4 growth hormone-secreting adenomas) in the OETA group and 4 patients (2 prolactinomas and 2 growth hormone-secreting adenomas) in the BETA group presented a hypersecretion-related syndrome, whereas a single axis defect or multiple axes defect were disclosed in 4 patients in the OETA group and 5 patients in the BETA group.

Preoperative visual examination revealed 8 patients in the OETA group and 12 patients in the BETA group with the visual defect.

The details of the basic characteristics and clinical symptoms of the patients were shown in Table 1. No significant difference was found between the two groups in these characteristics.

### Tumor removal

The GTR rate was 68.97% (20/29) for the OETA group and 67.74% (21/31) for the BETA group (determined by MRI which focused on the sellar region, including coronal and sagittal views—with native and contrast-enhanced sequences postoperatively).

The GTR was achieved in all PAs with Knosp grade 0–2 in the OETA group and 97.37% (15/16) in the BETA group. In the OETA group, subtotal resection (>70%) was achieved in 9 patients (Knosp grade 3: *n* = 4; Knosp grade 4: *n* = 5). In the BETA group, 9 patients received subtotal resection (Knosp grade 3: *n* = 3; Knosp grade 4: *n* = 6).

**Figure 3:**
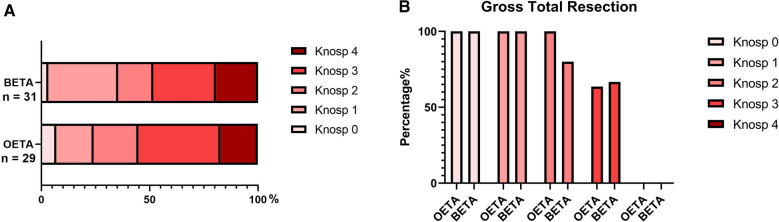
The tumor removal of the patients. (**A**) The constituent of patients with different Knosp grades in the two groups. (**B**) The gross total resection rate of tumors with different Konsp scores. binostril endoscopic transsphenoidal approach, BETA; one-and-a-half nostril endoscopic transsphenoidal approach, OETA.

No significant difference was found between the two groups regarding the tumor resection rate. The details of the tumor removal results were shown in Figure 3 and Supplementary Table S2.

### Post-operative endocrinal outcome

The hormonal remission rate of the functioning adenomas in the OETA group was 57.14% (4/7) and 75.00% (3/4) in the BETA group.

In the OETA group, of the 4 patients presenting pre-operative hypopituitarism, 3 patients improved while one patient remained unchanged. One patient developed new-onset hypocortisolism and two patients developed new-onset hypothyroidism post-operatively.

In the BETA group, of 5 patients presenting pre-operative hypopituitarism, 2 patients improved while 3 patients remained unchanged. One patient developed new-onset hypocortisolism and two patients developed new-onset hypothyroidism post-operatively.

No significant difference was found between the two groups regarding the endocrinal outcomes. The details of the endocrinal outcome were shown in Table 2 and Supplementary Table S2.

**Table 2:** Post-op endocrinal outcomes.

		OETA	BETA
Hormone remission, *n*/*N* = Functioning adenoma		4/7 (57.14%)	3/4 (75.00%)
Patient with pre-op hypopituitarism, *n*/*N* = pre-op hypopituitarism	Improved	3/4 (75.00%)	2/5 (40.00%)
	Unchanged	1/4 (25.00%)	3/5 (60.00%)
New onset hypopituitarism, *n*	Hypocortisolism	1	1
	Hypothyroidism	2	2

### Post-operative visual outcome

Visual defect improved in 5 cases (5/8, 62.50%) in the OETA group and 7 cases (7/12, 58.33%) in the BETA group. None of the patients of both groups with a normal preoperative visual assessment experienced any postoperative worsening.

No significant difference was found between the two groups regarding the visual outcomes. The details of the visual outcome were shown in Table 3 and Supplementary Table S2.

**Table 3:** Post-op visual outcomes.

		OETA	BETA
Patient with pre-op visual defect, *n*/*N* = pre-op visual defect	Improved	5/8 (62.50%)	7/12 (58.33%)
	Unchanged	3/8 (37.50%)	5/12 (41.67%)
	Worsened	0/8 (0%)	0/12 (0%)
Patient with normal pre-op visual function, *n*/*N* = normal pre-op visual function	Unchanged	21/21 (100%)	19/19 (100%)
	Worsened	0/21 (0%)	0/21 (0%)

### Surgical complications

The most common surgical complication was temporary diabetes insipidus in this series, which occurred in 6 patients in OETA and 5 patients in BETA. All these patients with temporary diabetes insipidus recovered after three months. Concerning the postoperative CSF leakage, the number was 2 in OETA and 3 in BETA. Of the 5 patients, intracranial infection (2 in OETA and 2 in BETA) occurred in 4 of them. After antibiotic treatment and lumbar drainage, all of them recovered with no sequela. Two patients reported in BETA while none occurred in OETA.

No carotid injury or cranial nerve injury was observed. There was no death related to the procedure.

No significant difference was found between the two groups regarding the surgical complication. The details of the surgical complication were shown in Table 4 and Supplementary Table S2.

**Table 4:** Postoperative complication.

Postoperative complication, *n*	OETA (*n* = 29)	BETA (*n* = 31)
Carotid injury	0 (0%)	0 (0%)
CSF leakage	2 (6.90%)	3 (9.68%)
Intracranial infection	2 (6.90%)	2 (6.45%)
Cranial nerve injury	0 (0%)	0 (0%)
Temporary diabetes insipidus	6 (20.69%)	5 (16.13%)
Nasal bleeding	0 (0%)	2 (6.45%)

### Sinonasal quality of life

We compared the results of ASK Nasal-12 between the OETA group and the BETA group pre-operatively and post-operatively. The results were shown in Table 5. We found that patients in the OETA group complained less about dried nasal material at the 2-week point (*P* = 0.017) and reported better olfactory function at the 1-month point (*P* = 0.019) compared with the BETA group. However, there was no significant difference in early and long-term post-operative sinonasal QoL between the two approaches according to the entire ASK Nasal-12 metric.

**Table 5:** Comparison of ASK nasal-12 between OETA and BETA.

	preoperative	2-week postoperative	1-month postoperative	3-month postoperative	6-month postoperative	12-month postoperative	Long term postoperative
Sense of smell	0.45 ±** **0.99	3.10 ±** **0.31	2.55 ±** **0.78*	0.34 ±** **1.05	0.31 ±** **0.93	0.31 ±** **0.93	0.25 ±** **0.74
	0.55 ±** **1.06	3.10 ±** **0.30	2.97 ±** **0.48*	0.39 ±** **1.20	0.32 ±** **1.01	0.26 ±** **0.82	0.30 ±** **0.67
Sense of taste	0.24 ±** **0.83	1.14 ±** **0.58	1.07 ±** **0.59	0.14 ±** **0.74	0.10 ±** **0.56	0.10 ±** **0.56	0.13 ±** **0.61
	0.45 ±** **0.85	1.06 ±** **0.36	1.00 ±** **0.00	0.00 ±** **0.00	0.00 ±** **0.00	0.00 ±** **0.00	0.00 ±** **0.00
Urge to blow nose	0.55 ±** **1.15	1.10 ±** **0.41	1.03 ±** **0.19	0.00 ±** **0.00	0.00 ±** **0.00	0.00 ±** **0.00	0.08 ±** **0.28
	0.48 ±** **0.89	1.00 ±** **0.00	0.97 ±** **0.18	0.00 ±** **0.00	0.00 ±** **0.00	0.00 ±** **0.00	0.07 ±** **0.27
Postnasal discharge	0.21 ±** **0.56	1.48 ±** **0.95	0.39 ±** **0.79	0.00 ±** **0.00	0.00 ±** **0.00	0.00 ±** **0.00	0.00 ±** **0.00
	0.35 ±** **0.76	1.23 ±** **0.72	0.19 ±** **0.60	0.00 ±** **0.00	0.00 ±** **0.00	0.00 ±** **0.00	0.07 ±** **0.27
Thick nasal discharge	0.21 ±** **0.49	1.10 ±** **0.49	0.10 ±** **0.31	0.00 ±** **0.00	0.00 ±** **0.00	0.00 ±** **0.00	0.00 ±** **0.00
	0.26 ±** **0.68	1.13 ±** **0.43	0.00 ±** **0.00	0.00 ±** **0.00	0.00 ±** 0**.00	0.00 ±** 0**.00	0.11 ±** 0**.42
Headache	1.17 ±** **1.63	1.31 ±** **0.89	0.41 ±** **1.09	0.21 ±** **0.77	0.17 ±** **0.66	0.34 ±** **0.61	0.33 ±** **0.48
	1.03 ±** **1.40	1.13 ±** **0.67	0.16 ±** **0.58	0.16 ±** **0.58	0.10 ±** **0.40	0.13 ±** **0.43	0.30 ±** **0.72
Nose whistling	0.55 ±** **1.24	3.21 ±** **0.49	0.24 ±** **0.91	0.10 ±** **0.56	0.03 ±** **0.19	0.07 ±** **0.26	0.04 ±** **0.20
	0.58 ± 0.89	3.03 ±** **0.18	0.10 ±** **0.54	0.03 ±** **0.18	0.03 ±** **0.18	0.03 ±** **0.18	0.00 ±** **0.00
Dried nasal material	0.00 ±** **0.00	1.69 ±** **1.31*	0.76 ±** **0.91	0.00 ±** **0.00	0.00 ±** **0.00	0.00 ±** **0.00	0.08 ±** **0.28
	0.00 ±** **0.00	2.48 ±** **0.77*	0.84 ±** **0.93	0.00 ±** **0.00	0.00 ±** **0.00	0.03 ±** **0.18	0.00 ±** **0.00
Trouble breathing: day	0.31 ±** **0.71	3.03 ±** **0.19	1.31 ±** **0.60	0.28 ±** **0.65	0.10 ±** **0.56	0.10 ±** **0.41	0.08 ±** **0.41
	0.52 ±** **0.93	3.06 ±** **0.25	1.16 ±** **0.64	0.06 ±** **0.25	0.10 ±** **0.40	0.13 ±** **0.43	0.15 ±** **0.36
Trouble breathing: night	0.59 ±** **1.15	3.10 ±** **0.31	0.48 ±** **1.24	0.31 ±** **0.81	0.34 ±** **0.94	0.34 ±** **0.86	0.21 ±** **0.59
	0.87 ±** **1.18	3.06 ±** **0.25	0.19 ±** **0.79	0.06 ±** **0.25	0.06 ±** **0.25	0.03 ±** **0.18	0.04 ±** **0.19
Trouble breathing	0.34 ±** **0.94	3.00 ±** **0.00	0.17 ±** **0.60	0.00 ±** **0.00	0.00 ±** **0.00	0.00 ±** **0.00	0.00 ±** **0.00
	0.35 ±** **0.71	3.03 ±** **0.18	0.16 ±** **0.64	0.10 ±** **0.54	0.10 ±** **0.54	0.13 ±** **0.56	0.00 ±** **0.00

The upper line of the cell represents OETA and the lower line represents BETA.

* *P* < 0.05.

### Olfactory outcomes

**Figure 4:**
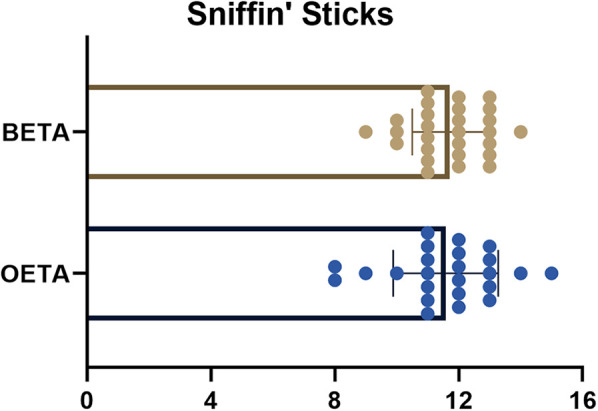
The scores of Sniffin’ Sticks in OETA and BETA. binostril endoscopic transsphenoidal approach, BETA; one-and-a-half nostril endoscopic transsphenoidal approach, OETA.

The mean score of the Sniffin’ Sticks odor identification test was 11.58 ± 1.69 in the OETA group and 11.70 ± 1.20 in the BETA group. There was no significant difference between the two groups (Figure 4).

## Discussion

The endonasal transsphenoidal approach was developed in the 1910s under the leadership of Oskar Hirsch, who never stopped advocating for this approach in the pre-antibiotic era (18). However, with the drawbacks of poor illumination and limited visualization, it was not until the introduction of the microscope in the 1960s that the microscopic transsphenoidal approach regained widespread favor (19). In the mid-1990s, a fully endoscopic endonasal transsphenoidal approach was reported and underwent dramatic evolution in the last two decades (20). Compared with microscopic surgery, endoscopic surgery provides a wider visual field and better illumination for sellar regions (21).

Some neurosurgeons are used to reach the sphenoid sinus through one nostril (the mononostril approach) with the help of a nasal speculum (21). However, the speculum restricts the bimanual handling of instruments (22). Another surgical technique, the binostril approach, typically does not require a nasal speculum and offers better maneuverability of instruments than the mononostril approach (21). In our meta-analysis comparing the mononostril approach and binostril approach for PAs, the binostril approach had a shorter length of hospital stay and fewer surgical complications (diabetes insipidus and hypopituitarism) than the mononostril approach while patients undergone the binostril approach tended to have a higher rate of epistaxis than the mononostril group (23).

With the use of the nasal flap for skull base reconstruction (24), the most common complication of EETA, the CSF leakage, has been controlled at a low rate. However, harvesting of this vascularized flap involves cutting the mucosa and is associated with an increased risk of postoperative nasal crusting, raising concern about worsening postoperative sinonasal quality of life (25). Among the nasal symptoms, the loss of olfaction was a major concern for surgeons and patients. In our previous review, the incidence of postoperative decreased olfactory function was 18.48% for the patients after endonasal skull base surgery (7). More attention should be paid to patients’ sinonasal QoL and olfaction.

To improve patients’ post-operative sinonasal QoL, we introduced OETA in 2016 (9). This technique provides not only a sufficient surgical corridor for a 2-surgeon/4-hands operation but also ensures minimal invasion to the nasal cavity, which combines the advantages of the binostril approach and the mononostril approach. In the technical report, we describe the procedures of OETA in detail and analyzed the clinical outcomes of 57 consecutive patients who underwent OETA between March 2014 and June 2015 at Jinling hospital (9). The GTR rate was 79% (9) for all the PAs. Post-operative hormone remission was achieved in 77.8% (14/18) of patients with functioning Pas (9). Concerning the sinonasal QoL, the most frequent complaint at the 2-week point was thick nasal discharge (36%), followed by loss of smell (28%) and trouble breathing during the day (18%) (9). Other symptoms, including post nasal discharge (8%), dried nasal material (6%), headache (6%), and decrease in sense of taste (4%) were also reported (9). Three months after surgery, most of the symptoms disappeared or were significantly relieved (9). The above results showed that the OETA was a simple and reliable technique.

We also compared surgical freedom and working angles between OETA and BETA in cadaveric dissection (10). The results showed that the OETA had similar surgical freedom and working angles to the BETA for most anatomic targets in the sellar or parasellar region.

A prospective randomized controlled trial was then registered to provide high-quality evidence for this approach. After two years of enrollment and several years of follow-up, the results confirmed that the two approaches had similar GTR rates (OETA: 68.97%; BETA: 67.74%). As to the invasive PAs, the GTR rates were also similar between the two groups (OETA: 43.75%; BETA: 40.00%).

Three patients with prolactinomas (2 in OETA and 1 in BETA) did not reach hormone remission after surgery, with 2 Knosp grade 4 PAs and 1 Knosp grade 3 PA. Dopamine receptor agonist therapy was taken for them to control the serum prolactin level. With regards to the 6 patients with acromegaly, we luckily achieved GTR in all of them and the patients reached hormone remission post-operatively, probably because there was no Knosp grade 4 PA in these 6 patients. New-onset Hypopituitarism occurred in 6 patients (OETA: *n* = 3; BETA: *n* = 3) and they were transferred to endocrinologists for hormone replacement therapy.

There was also no significant difference regarding the surgical complications between OETA and BETA. Eleven patients (OETA: *n* = 6; BETA: *n* = 5) suffered from temporary diabetes insipidus post-operatively and all recovered three months after surgery. The most worrisome complication, postoperative CSF leakage, occurred in five patients (OETA: *n* = 2; BETA: *n* = 3) and consequent intracranial infection occurred in four of them (OETA: *n* = 2; BETA: *n* = 2). After antibiotic treatment and lumbar drainage, all of them recovered with no sequela. It’s also worth noting that two patients reported epistaxis in BETA while none reported in OETA. No carotid injury or cranial nerve injury was observed in these patients.

As for the sinonasal quality of life, the patients in the two groups had the same recovery course. Although OETA seemed to have potential benefits in two components of ASK Nasal-12 at early postoperative time points (dried nasal material at the 2-week point, *P* = 0.017; olfaction at the 1-month point, *P* = 0.019), there was no significant difference in early and long-term post-operative sinonasal QoL between the two approaches according to the entire ASK Nasal-12 metric.

Many factors may affect sinonasal outcomes after endoscopic endonasal surgery, including the intraoperative protection of nasal mucosa, harvesting nasoseptal flaps, nasal packing, and postoperative nasal care. The harvest of the nasoseptal flap is an important factor that is associated with worse sinonasal QoL within the early postoperative period (26). In our series, 9 patients received nasoseptal flaps in skull base reconstruction (4 in OETA and 5 in BETA).

More preservation of the nasal natural structures is a feasible way to improve the sinonasal QoL. Several groups have reported alternative closure of the skull base to the nasoseptal flap with the local sphenoidal mucosa or the sellar floor flap (27, 28), which could help improve sinonasal outcomes.

The reason why this trial failed to find a difference between the two approaches in sinonasal outcomes may lie in the small numbers of the trial and the relatively small difference between the two approaches.

We assessed patients’ olfactory performance objectively with the Sniffin’ Sticks. Both two groups had satisfactory results from objective olfactory examinations (OETA: 11.58 ± 1.69; BETA: 11.70 ± 1.20). 54.17% (13/24) of patients in OETA had a great olfactory assessment score (>11) and the rate in BETA was 55.56% (15/27).

## Conclusion

In this single tertiary center trial, the results proved that the OETA achieved the same surgical outcomes and post-operative sinonasal QoL as the BETAL.

## Data Availability

The original contributions presented in the study are included in the article/**Supplementary Material**, further inquiries can be directed to the corresponding author/s.
