# Supplementary material for: One-and-a-half nostril versus binostril endoscopic transsphenoidal approach to the pituitary adenomas: A prospective randomized controlled trial
Source: Front Surg. 2022 Sep 23;9:1007883. doi: 10.3389/fsurg.2022.1007883 (PMC9632956; doi:10.3389/fsurg.2022.1007883)
Supplement: Supplementary file 1 [file Datasheet1.docx]

Supplemental Table 1

**Anterior Skull Base Nasal Inventory-12 (ASK Nasal-12)**

| **Anterior Skull Base Nasal Inventory-12**  **Evaluator’s name: _________________________________** | | | | | | |
| --- | --- | --- | --- | --- | --- | --- |
|  | no problem  (0) | very mild  Problem  (1) | mild  Problem  (2) | moderate  problem  (3) | Severe problem  (4) | very severe problem  (5) |
| sense of smell |  |  |  |  |  |  |
| sense of taste |  |  |  |  |  |  |
| urge to blow nose |  |  |  |  |  |  |
| postnasal discharge |  |  |  |  |  |  |
| thick nasal discharge |  |  |  |  |  |  |
| headache |  |  |  |  |  |  |
| nose whistling |  |  |  |  |  |  |
| dried nasal material |  |  |  |  |  |  |
| trouble breathing: day |  |  |  |  |  |  |
| trouble breathing: night |  |  |  |  |  |  |
| trouble breathing |  |  |  |  |  |  |
| overall nasal functioning |  |  |  |  |  |  |

The question is scored on a 6-point Likert scale (0–5). Higher scores indicate worse conditions of items.

Supplemental Table 2 The details of the patients

| Patient ID | Group | Sex | Age | Course of disease (month) | Tumour Type | Maximum diameter | Knosp grade | Follow-up (month) | Tumor Resection | Hormone remission | Pre-op endocrinal symptoms | Post-op endocrinal outcome | Pre-op visual symptoms | Post-op visual outcome | Surgical complications |
| --- | --- | --- | --- | --- | --- | --- | --- | --- | --- | --- | --- | --- | --- | --- | --- |
| Patient 01 | OETA | Male | 21 | 60 | Prolactinoma | 4.9 | 3 | 24 | Subtotal resection | No | Hyperprolactinemia, Panhypopituitarism | Unchanged | Impaired vision, Bitemporal hemianopia | Unchanged | temporary diabetes insipidus |
| Patient 02 | OETA | Male | 61 | 120 | GH-secreting adenoma | 1.9 | 2 | 31 | Total resection | Yes | / | Remission | / | Unchanged (Normal) | temporary diabetes insipidus |
| Patient 03 | OETA | Female | 12 | 8 | Prolactinoma | 5 | 3 | 27 | Subtotal resection | No | Hyperprolactinemia, Panhypopituitarism | Unchanged | Impaired vision, Bitemporal hemianopia | Unchanged | temporary diabetes insipidus |
| Patient 04 | OETA | Male | 50 | 6 | Nonfunctioning adenoma | 2.1 | 2 | 22 | Total resection | / | / | / | / | Unchanged (Normal) | / |
| Patient 05 | OETA | Male | 60 | 0.3 | Nonfunctioning adenoma | 3.8 | 4 | 25 | Subtotal resection | / | Hypocortisolism, Hypogonadism | Improved | / | Unchanged (Normal) | / |
| Patient 06 | OETA | Female | 38 | 72 | Nonfunctioning adenoma | 0.8 | 0 | 21 | Total resection | / | / | / | / | Unchanged (Normal) | / |
| Patient 07 | OETA | Male | 74 | 24 | Nonfunctioning adenoma | 2.2 | 3 | 19 | Total resection | / | / | / | Impaired vision, Left temporal hemianopia | Improved | / |
| Patient 08 | OETA | Female | 47 | 1 | GH-secreting adenoma | 1.6 | 3 | 23 | Total resection | Yes | Acromegaly | Remission, Hypocortisolism (new onset) | / | Unchanged (Normal) | / |
| Patient 09 | OETA | Female | 51 | 36 | Nonfunctioning adenoma | 1.6 | 4 | 26 | Subtotal resection | / | / | / | / | Unchanged (Normal) | / |
| Patient 10 | OETA | Female | 54 | 12 | Nonfunctioning adenoma | 1.8 | 4 | 18 | Subtotal resection | / | / | / | Impaired vision, Right temporal hemianopia | Improved | CSF leakage, intracranial infection |
| Patient 11 | OETA | Male | 39 | 60 | GH-secreting adenoma | 2.7 | 3 | 20 | Total resection | Yes | Acromegaly | Remission | / | Unchanged (Normal) | CSF leakage, intracranial infection, temporary diabetes insipidus |
| Patient 12 | OETA | Male | 42 | 6 | Prolactinoma | 4 | 4 | 25 | Subtotal resection | No | Hyperprolactinemia | Improved | Impaired vision, Bitemporal hemianopia | Unchanged | / |
| Patient 13 | OETA | Female | 29 | 11 | Nonfunctioning adenoma | 2 | 3 | 28 | Total resection | / | / | / | / | Unchanged (Normal) | / |
| Patient 14 | OETA | Male | 60 | 1 | Nonfunctioning adenoma | 3.1 | 3 | 26 | Total resection | / | / | / | / | Unchanged (Normal) | / |
| Patient 15 | OETA | Female | 50 | 12 | GH-secreting adenoma | 1.4 | 3 | 18 | Total resection | Yes | Acromegaly | Remission | / | Unchanged (Normal) | / |
| Patient 16 | OETA | Male | 54 | 24 | Nonfunctioning adenoma | 1.4 | 4 | 13 | Subtotal resection | / | / | / | / | Unchanged (Normal) | / |
| Patient 17 | OETA | Male | 52 | 6 | Nonfunctioning adenoma | 3.5 | 3 | 19 | Subtotal resection | / | / | / | Impaired vision, Left temporal hemianopia | Improved | / |
| Patient 18 | OETA | Female | 51 | 60 | Nonfunctioning adenoma | 1.8 | 2 | 12 | Total resection | / | / | / | / | Unchanged (Normal) | / |
| Patient 19 | OETA | Male | 59 | 0.2 | Nonfunctioning adenoma | 1.5 | 1 | 21 | Total resection | / | / | Hypothyroidism (new onset) | / | Unchanged (Normal) | temporary diabetes insipidus |
| Patient 20 | OETA | Male | 59 | 0.5 | Nonfunctioning adenoma | 2.5 | 2 | 14 | Total resection | / | / | / | / | Unchanged (Normal) | / |
| Patient 21 | OETA | Male | 47 | 60 | Nonfunctioning adenoma | 2.3 | 2 | 23 | Total resection | / | / | / | / | Unchanged (Normal) | / |
| Patient 22 | OETA | Male | 28 | 36 | Nonfunctioning adenoma | 2.4 | 3 | 21 | Subtotal resection | / | / | / | / | Unchanged (Normal) | / |
| Patient 23 | OETA | Female | 48 | 36 | Nonfunctioning adenoma | 2 | 1 | 17 | Total resection | / | / | / | Impaired vision | Improved | temporary diabetes insipidus |
| Patient 24 | OETA | Female | 49 | 1 | Nonfunctioning adenoma | 3 | 2 | 18 | Total resection | / | / | / | / | Unchanged (Normal) | / |
| Patient 25 | OETA | Female | 70 | 12 | Nonfunctioning adenoma | 3 | 1 | 17 | Total resection | / | / | Hypothyroidism (new onset) | / | Unchanged (Normal) | / |
| Patient 26 | OETA | Female | 49 | 120 | Nonfunctioning adenoma | 0.8 | 0 | 20 | Total resection | / | / | / | / | Unchanged (Normal) | / |
| Patient 27 | OETA | Male | 68 | 36 | Nonfunctioning adenoma | 3 | 1 | 14 | Total resection | / | Hypocortisolism | Improved | Impaired vision, Left temporal hemianopia | Improved | / |
| Patient 28 | OETA | Female | 60 | 0.5 | Nonfunctioning adenoma | 1.2 | 1 | 18 | Total resection | / | / | / | / | Unchanged (Normal) | / |
| Patient 29 | OETA | Female | 50 | 48 | Nonfunctioning adenoma | 1.3 | 3 | 13 | Total resection | / | / | / | / | Unchanged (Normal) | / |
| Patient 30 | BETA | Female | 67 | 0.2 | Nonfunctioning adenoma | 5 | 4 | 27 | Subtotal resection | / | Panypopituitarism | Unchanged | Impaired vision, Bitemporal hemianopia | Unchanged | / |
| Patient 31 | BETA | Female | 63 | 2 | Prolactinoma | 3 | 2 | 26 | Total resection | Yes | Hyperprolactinemia | Remission | Impaired vision, Bitemporal hemianopia | Improved | / |
| Patient 32 | BETA | Male | 51 | 0.2 | Nonfunctioning adenoma | 1.9 | 4 | 24 | Subtotal resection | / | Hypocortisolism | Improved | / | Unchanged (Normal) | temporary diabetes insipidus |
| Patient 33 | BETA | Female | 63 | 0.5 | Nonfunctioning adenoma | 1.5 | 1 | 27 | Total resection | / | / | / | / | Unchanged (Normal) | / |
| Patient 34 | BETA | Female | 47 | 12 | Nonfunctioning adenoma | 2 | 3 | 23 | Total resection | / | / | / | / | Unchanged (Normal) | / |
| Patient 35 | BETA | Female | 48 | 18 | Nonfunctioning adenoma | 2.6 | 4 | 14 | Subtotal resection | / | / | Hypocortisolism (new onset) | Impaired vision, Right temporal hemianopia | Improved | CSF leakage, intracranial infection |
| Patient 36 | BETA | Female | 38 | 120 | Nonfunctioning adenoma | 1.1 | 3 | 23 | Total resection | / | / | / | / | Unchanged (Normal) | CSF leakage |
| Patient 37 | BETA | Male | 51 | 0.5 | Nonfunctioning adenoma | 2.4 | 3 | 21 | Subtotal resection | / | / | / | / | Unchanged (Normal) | nepistaxis |
| Patient 38 | BETA | Male | 20 | 0.5 | Nonfunctioning adenoma | 1.6 | 1 | 25 | Total resection | / | / | / | / | Unchanged (Normal) | / |
| Patient 39 | BETA | Male | 43 | 0.5 | Nonfunctioning adenoma | 1.6 | 1 | 18 | Total resection | / | / | / | / | Unchanged (Normal) | temporary diabetes insipidus |
| Patient 40 | BETA | Female | 69 | 12 | Nonfunctioning adenoma | 3 | 2 | 19 | Total resection | / | / | / | Impaired vision, Bitemporal hemianopia | Improved | temporary diabetes insipidus |
| Patient 41 | BETA | Male | 63 | 24 | Nonfunctioning adenoma | 3.7 | 1 | 23 | Total resection | / | / | / | Impaired vision, Bitemporal hemianopia | Unchanged | / |
| Patient 42 | BETA | Male | 44 | 2 | GH-secreting adenoma | 1.4 | 3 | 22 | Total resection | Yes | Acromegaly | Remission |  | Unchanged (Normal) | / |
| Patient 43 | BETA | Female | 54 | 6 | Nonfunctioning adenoma | 1.8 | 1 | 20 | Total resection | / | / | / | Impaired vision | Improved | / |
| Patient 44 | BETA | Male | 49 | 24 | Nonfunctioning adenoma | 4 | 2 | 15 | Subtotal resection | / | Hypocortisolism, Hypogonadism | Unchanged | Impaired vision, Left temporal hemianopia | Unchanged | / |
| Patient 45 | BETA | Male | 44 | 0.3 | Nonfunctioning adenoma | 2 | 1 | 21 | Total resection | / | / | / |  | Unchanged (Normal) | / |
| Patient 46 | BETA | Female | 47 | 36 | Nonfunctioning adenoma | 4.4 | 4 | 12 | Subtotal resection | / | Panhypopituitarism | Unchanged | Impaired vision, Right temporal hemianopia | Unchanged | CSF leakage, intracranial infection |
| Patient 47 | BETA | Female | 46 | 60 | GH-secreting adenoma | 3 | 3 | 26 | Total resection | Yes | Acromegaly | Remission, Hypothyroidism (new onset) | / | Unchanged (Normal) | / |
| Patient 48 | BETA | Female | 46 | 4 | Nonfunctioning adenoma | 3 | 4 | 22 | Subtotal resection | / | / | / | Impaired vision | Improved | / |
| Patient 49 | BETA | Male | 60 | 1 | Nonfunctioning adenoma | 1.8 | 1 | 22 | Total resection | / | / | / | / | Unchanged (Normal) | / |
| Patient 50 | BETA | Female | 50 | 1 | Nonfunctioning adenoma | 2 | 1 | 23 | Total resection | / | / | / | / | Unchanged (Normal) | / |
| Patient 51 | BETA | Female | 47 | 1 | Nonfunctioning adenoma | 2 | 3 | 18 | Subtotal resection | / | / | / | / | Unchanged (Normal) | temporary diabetes insipidus |
| Patient 52 | BETA | Male | 58 | 0.2 | Nonfunctioning adenoma | 0.7 | 0 | 19 | Total resection | / | / | / | / | Unchanged (Normal) | / |
| Patient 53 | BETA | Female | 46 | 120 | Nonfunctioning adenoma | 1.4 | 2 | 22 | Total resection | / | / | / | / | Unchanged (Normal) | / |
| Patient 54 | BETA | Male | 50 | 1 | Nonfunctioning adenoma | 1 | 1 | 28 | Total resection | / | / | Hypothyroidism (new onset) | / | Unchanged (Normal) | / |
| Patient 55 | BETA | Male | 33 | 2 | Nonfunctioning adenoma | 2.3 | 3 | 20 | Total resection | / | Hypocortisolism | Improved | / | Unchanged (Normal) | / |
| Patient 56 | BETA | Female | 52 | 0.5 | Nonfunctioning adenoma | 3 | 2 | 18 | Total resection | / | / | / | / | Unchanged (Normal) | / |
| Patient 57 | BETA | Male | 66 | 24 | Nonfunctioning adenoma | 3.4 | 3 | 20 | Subtotal resection | / | / | / | Impaired vision, Bitemporal hemianopia | Improved | / |
| Patient 58 | BETA | Male | 46 | 1 | Prolactinoma | 4 | 4 | 13 | Subtotal resection | No | Hyperprolactinemia | Unchanged | Impaired vision, Bitemporal hemianopia | Unchanged | temporary diabetes insipidus |
| Patient 59 | BETA | Male | 58 | 36 | Nonfunctioning adenoma | 3 | 3 | 20 | Total resection | / | / | / | Impaired vision, Right temporal hemianopia | Improved | / |
| Patient 60 | BETA | Male | 59 | 0.7 | Nonfunctioning adenoma | 1.7 | 1 | 21 | Total resection | / | / | / | / | Unchanged (Normal) | nepistaxis |
