# Supplementary material for: One-and-a-half nostril versus binostril endoscopic transsphenoidal approach to the pituitary adenomas: A prospective randomized controlled trial
Source: Front Surg. 2022 Sep 23;9:1007883. doi: 10.3389/fsurg.2022.1007883 (PMC9632956; doi:10.3389/fsurg.2022.1007883)
Supplement: Supplementary file 2 [file Datasheet2.docx]

**ChiCTR-IOR-16008222**

**date_registration: 2016-04-05**

**primary_sponsor: Department of Neurosurgery, Jinling Hospital, School of Medicine, Nanjing University**

**public_title: One and a half nostril versus binostril endoscopic transsphenoidal approach for pituitary adenomas**

**date_enrolment: 2016-04-05**

**target_size: Binostril endoscopic transsphenoidal approach for pituitary adenomas:30; One and a nostril endoscopic transsphenoidal approach for pituitary adenoma:30**

**study_type: Interventional study**

**affiliation: Jinling Hospital, School of Medicine, Nanjing University**

**address: 305 East Zhongshan Road, Nanjing, China**

**telephone: +86 13401931231**

**email: machiyuan_nju@126.com**

**inclusion_criteria:**

**1)The participators sign the consent form of this study voluntarily;**

**2) Age greater than or equal to 18 years old, less than or equal to 80 years old, both male and female, and patients with pituitary adenoma;**

**3) Without central infection and severe systemic infection;**

**4) No blood coagulation disorders, and no severe liver and kidney impairment;**

**5) Not pregnancy, nursing mothers.</inclusion_criteria>**

**exclusion_criteria：**

**1) The anesthesiologist determine patients doesn't fit to the researche;**

**2) General condition is poor, patients cannot objectively describe the symtom, or have serious infections, respiratory disturbance, or cannot cooperate actively;**

**3) Intracranial tumor metastasis patients with disturbance of consciousness;**

**4) Central infection, severe systemic infection;**

**5) Blood coagulation disorders; Patients with severe liver and kidney impairment: general for twice the normal value;**

**6）women in gestational or lactational period**

**7) Directly involved in the research of the sponsor or researchers or their family members;**

**8) The researchers think that there is any reason not spots.**

**primary_outcome**

**Gross tumor resection; Hormonal remission rate; Complications**

**secondary_outcome**

**the sinonasal quality of life; the olfactory outcomes**

**intervention**

**OETA : The patient was under general anesthesia and in supine position with 10 degrees of extension. The bilateral nasal cavities were packed with cottonoids containing 0.01% epinephrine for vasoconstriction and irrigated with iodine for disinfection. The operation started from the right nostril under a 0° endoscope (Karl Storz, Tuttlingen, Germany). The right inferior and middle turbinates were out-fractured for access to the sphenoethmoidal recess. Then, the right “rescue” nasoseptal flap was made with caution to protect the olfactory epithelium. The next step was to incise the left nasal septal mucosa with a vertical incision which extended approximately 2 cm at the anterior-level of the middle turbinate. These procedures provided sufficient binasal access for two surgeons using the four-handed technique. The sphenoid ostium was then identified and enlarged with a low-speed drill and rongeur. After enlargement of the sphenoid ostia and removal of the sphenoid septum, the following procedures were the same as the BETA.**

**BETA : The nasal preparation and the creation of “rescue” nasoseptal flap was done the same way as the OETA. Sphenoidotomy was performed on both sides. A posterior septal window was created by removing the posterior part of the bony nasal septum to allow bilateral access. The optic nerve canal and the carotid prominence were identified as landmarks. The sellar floor was then flattened with a drill and opened with the rongeur. After opening the dura with scissors, the tumor was removed with slow movements using curettes and suction cannula. The 30°-angled endoscope was introduced into the sella for inspection of tumor remnants. If intraoperative cerebrospinal fluid (CSF) leak occurred, the unilateral “rescue” nasoseptal flap was then fashioned to ensure a vascularized repair. Gelfoam and Tabotamp fibrillar (Johnson & Johnson Medical GmbH) were routinely used for skull base reconstruction. Nasal packing was not used routinely.**

**Follow-up**

**The patients were asked to return to the hospital for radiologic examinations and hormone level checks. at 1, 3, 6, 12 months and long-term point (more than 16 months) postoperatively for determination of tumor resection and hormonal remission.**

**The questionnaire Anterior Skull Base Nasal Inventory-12 and Sniffin’ Sticks were distributed to participants to evaluate their sinonasal quality of life at 7 due time point (preoperatively, 2-week postoperatively, 1-month postoperatively, 3-month postoperatively, 6-month postoperatively, 12-month postoperatively, long-time postoperatively).**
